# Melatonin and Indole-3-Propionic Acid Reduce Oxidative Damage to Membrane Lipids Induced by High Iron Concentrations in Porcine Skin

**DOI:** 10.3390/membranes11080571

**Published:** 2021-07-29

**Authors:** Aleksandra Rynkowska, Jan Stępniak, Małgorzata Karbownik-Lewińska

**Affiliations:** 1 Department of Oncological Endocrinology, Medical University of Lodz, 90-752 Łódź, Poland; aleksandra.rynkowska@umed.lodz.pl (A.R.); jan.stepniak@umed.lodz.pl (J.S.); 2 Polish Mother’s Memorial Hospital—Research Institute, 93-338 Łódź, Poland

**Keywords:** skin, membrane lipids, melatonin, indole-3-propionic acid, Fenton reaction, lipid peroxidation, oxidative stress

## Abstract

Iron excess in tissues results in increased oxidative damage. Among different tissues, the skin can particularly be severely damaged by oxidative stress, as it is exposed not only to endogenous but also directly to exogenous pro-oxidants. The skin is especially vulnerable to harmful oxidative stress. Melatonin and indole-3-propionic acid (IPA), two indole substances, are efficient antioxidants. This study aims to evaluate the potential protective effects of melatonin and IPA against oxidative damage to membrane lipids (lipid peroxidation (LPO)), induced in porcine skin homogenates by the Fenton reaction (Fe^2+^ + H_2_O_2_ → Fe^3+^ + ^•^OH + OH^−^) when iron is used in extremely high concentrations. Skin homogenates were incubated in the presence of FeSO_4_ (2400, 1200, 600, 300, 150 and 75 µM) + H_2_O_2_ (5 mM) with/without melatonin or IPA. LPO level (MDA + 4-HDA/mg protein) was measured spectrophotometrically. Melatonin, in its highest used concentration (5.0 mM), prevented FeSO_4_ (1200 mM)-induced LPO, whereas it was effective in concentrations as low as 2.5 mM against all lower iron concentrations. IPA was protective in concentrations as low as 2.5 mM independently of FeSO_4_ concentration. In conclusion, melatonin and IPA effectively protect against oxidative damage to membrane lipids induced by high concentrations of iron in porcine skin; therefore, both can be considered pharmacological agents in the case of disorders associated with excessive iron accumulation in the skin.

## 1. Introduction

Biological membranes, being a source of polyunsaturated fatty acids (PUFAs), are susceptible to reactive oxygen species (ROS) attack. PUFAs are regulators of many (patho)physiological processes among others such as inflammation, immunity and cellular growth [1]. The overproduction of free radicals and their action on membrane PUFAs result in oxidative damage to membrane lipids (lipid peroxidation (LPO)). This causes structural changes in cellular membranes, impairing cellular functions and contributing to numerous diseases [2]. In agreement with this, some kinds of skin abnormalities are associated with increased oxidative stress [3,4]. Moreover, ROS contribute to skin aging [5].

Among different tissues, the skin can particularly be severely damaged by oxidative stress, as it is exposed not only to endogenous but also directly to a variety of exogenous pro-oxidants, such as environmental pollutants or UV radiation. We recently documented that increased levels of lipid peroxidation products in the epidermis results from smoking [6] or obesity [7]. Additionally, direct skin exposure to pro-oxidants, such as ultraviolet UVA and UVB radiation, enhanced oxidative damage of membrane lipids in both normal and psoriatic keratinocytes and fibroblasts [8].

The well-known reaction of oxidative stress is the Fenton reaction (Fe^2+^ + H_2_O_2_ → Fe^3+^ + ^•^OH + OH^−^). During this reaction, the most harmful free radical, the hydroxyl radical (^•^OH), is produced. The model of Fenton reaction-induced oxidative damage to macromolecules has been widely used in laboratories [9,10,11,12,13,14,15]. Both substrates of the Fenton reaction, i.e., iron, used as a ferrous ion (Fe^2+^), and hydrogen peroxide (H_2_O_2_) are natural and essential elements for the proper functioning of organisms, with iron serving as a micronutrient and (H_2_O_2_) being an important ROS indispensable for certain biochemical reactions. However, their excess increases oxidative stress, contributing to the process of carcinogenesis and many other diseases. Iron overload is typically attributed to hemochromatosis [16].

To offset the negative effects of oxidative damage, the human body obtains exogenous antioxidants from the diet (e.g., vitamins) or produces endogenous antioxidants themselves (e.g., hormones) [17]. One of the best known endogenous antioxidants and free radical scavengers is N-acetyl-5-methoxytryptamine (melatonin). It is a multifunctional molecule and a neurohormone, produced and released mainly by the pineal gland [18]. An age-associated decrease in melatonin production and in melatonin receptors in the skin results in weaker protection against exogenous pro-oxidants [19].

Similar in chemical structure to melatonin (possessing a heterocyclic aromatic ring) is indole-3-propionic acid (IPA), which is a deamination product of tryptophan. In the human body, IPA is produced by the human microbiota; its presence in cerebrospinal fluid results from bacterial metabolism in the intestine [20]. Whereas IPA is a known auxin, melatonin is documented to act synergistically with auxins in plants [21]. These two indole substances possess significant antioxidative properties, which was confirmed in numerous experimental or clinical studies [6,9,10,11,14,22,23].

As the presence of melatonin in the skin has been confirmed [24] and its protective action on the skin has been discussed [25], examining the effects of exogenous indole substances in this tissue is of importance.

The aim of this study was to evaluate the potential protective effects of melatonin and IPA against oxidative damage to membrane lipids induced by Fenton reaction substrates in porcine skin homogenates. Iron, one of the Fenton reaction substrates, was used in extremely high concentrations.

## 2. Materials and Methods

### 2.1. Chemicals

Melatonin, indole-3-propionic acid, ferrous sulfate (FeSO_4_) and hydrogen peroxide (H_2_O_2_) were purchased from Sigma (St. Louis, MO, USA). Ethanol (96%) was purchased from Stanlab (Lublin, Poland). The LPO-586 kit for LPO was obtained from Enzo Life Science (Farmingdale, NY, USA). All the used chemicals were of analytical grade and came from commercial sources. 

### 2.2. Animals

Porcine abdominal skin was collected from twenty-four (24) animals at a slaughterhouse, frozen on solid CO_2_ and stored at −80 °C until assay. 

### 2.3. Assay of Lipid Peroxidation

Skin tissue was homogenized in ice-cold 50 mM Tris-HCl buffer (pH 7.4) (10%, *w*/*v*) and then incubated for 30 min at 37 °C in the presence of examined substances. Melatonin and IPA were dissolved in absolute ethanol. The concentration of ethanol in the final incubation medium was 1% (*v*/*v*).

In the 1st experiment, homogenates were incubated in the presence of FeSO_4_ (2400, 1200, 600, 300, 150, 75, 37.5, 18.75, 9.375, 4.6875 and 2.34375 µM) + H_2_O_2_ (5 mM). 

In the 2nd experiment, homogenates were incubated in the presence of FeSO_4_ used in one of six different concentrations (2400, 1200, 600, 300, 150 and 75 µM) + H_2_O_2_ (5 mM) with or without melatonin (0.0, 5.0, 2.5, 1.0, 0.5, 0.25, 0.1, 0.01, 0.001, 0.0001 and 0.00001 mM). A melatonin concentration of 5 mM is the highest achievable in vitro concentration due to the limited solubility of this compound.

In the 3rd experiment, homogenates were incubated in the presence of FeSO_4_ used in one of six different concentrations (2400, 1200, 600, 300, 150 and 75 µM) + H_2_O_2_ (5 mM) with or without IPA (0.0, 7.5, 5.0, 2.5, 1.0, 0.5, 0.25, 0.1, 0.01, 0.001 and 0.0001 mM). An IPA concentration of 7.5 mM is the highest achievable in vitro concentration due to the limited solubility of this compound.

Each experiment was run in duplicate and repeated three times. 

### 2.4. Measurement of Lipid Peroxidation Products

The concentration of malondialdehyde + 4-hydroxyalkenals (MDA + 4-HDA), as an index of LPO, was measured in tissue homogenates (using a Ultrospec 2000 spectrophotometer, purchased from Amersham Pharmacia Biotech (Uppsala, Sweden)), as described elsewhere [11]. Protein was measured using the Bradford method [26].

### 2.5. Statistical Analyses

Data were statistically analyzed using a one-way analysis of variance (ANOVA), followed by the Student–Newman–Keuls test. Statistical significance was determined at the level of *p* < 0.05. Results are presented as the mean ± SE.

## 3. Results

**Figure 1:**
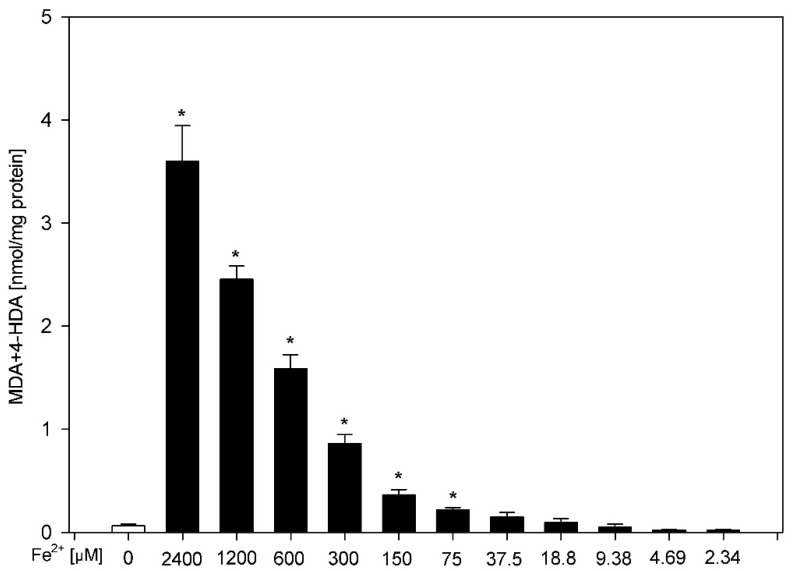
Concentration of malondialdehyde + 4-hydroxyalkenals (MDA + 4-HDA) in skin homogenates. Homogenates were incubated in the presence of FeSO_4_ (2400, 1200, 600, 300, 150, 75, 37.5, 18.75, 9.375, 4.6875 and 2.34375 μM) plus H_2_O_2_ (5 mM). Data are expressed as the amount of MDA + 4-HDA (nmol) per mg of protein. Bars represent the mean  ±  SE of three independent experiments run in duplicate. * *p* < 0.05 vs. control (white bar).

The incubation of skin homogenates in the presence of FeSO_4_ plus H_2_O_2_ (5 mM) resulted in a ferrous concentration-dependent increase (statistically significant for concentrations of 2400, 1200, 600, 300, 150 and 75 μM) in LPO level (Figure 1). These concentrations of FeSO_4_ were selected for subsequent experiments.

**Figure 2:**
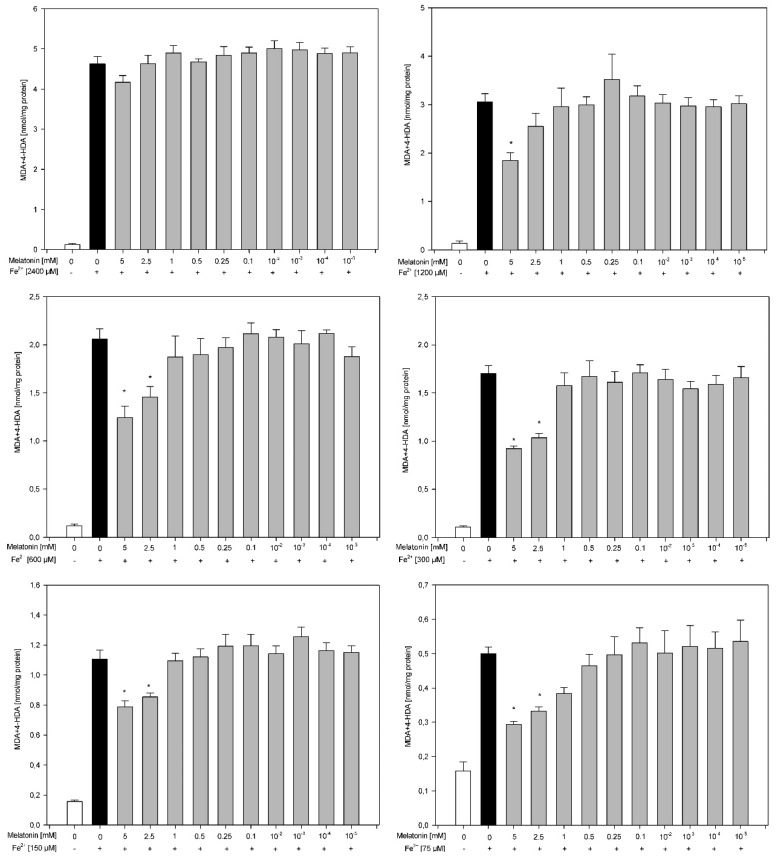
Concentration of malondialdehyde + 4-hydroxyalkenals (MDA + 4-HDA) in skin homogenates. Homogenates were incubated in the presence of FeSO_4_ used in one of six different concentrations (2400, 120, 600, 300, 150 and 75 µM) plus H_2_O_2_ (5 mM) and, additionally, in the presence of melatonin (0.0, 5.0, 2.5, 1.0, 0.5, 0.25, 0.1, 0.01, 0.001, 0.0001 and 0.00001 mM). Data are expressed as the amount of MDA + 4-HDA (nmol) per mg of protein. Bars represent the mean ± SE of three independent experiments run in duplicate. * *p* < 0.05 vs. respective concentration of Fe^2+^ (black bars). All bars representing either Fe^2+^ (black bars) or Fe^2+^ plus melatonin (gray bars) are significantly higher than bars representing control (white bars).

Results of the second experiment revealed that the protective effects of melatonin against experimentally induced LPO depend on the FeSO_4_ concentration and were observed for almost all concentrations of FeSO_4_ except one, i.e., 2400 μM. Against a FeSO_4_ concentration of 1200 μM, melatonin was effective only in the highest used concentration, namely 5.0 mM. In the case of lower concentrations of iron (600, 300, 150 and 75 μM), melatonin reduced the level of LPO when this indoleamine was used in the two highest concentrations, i.e., 5.0 and 2.5 mM (Figure 2).

**Figure 3:**
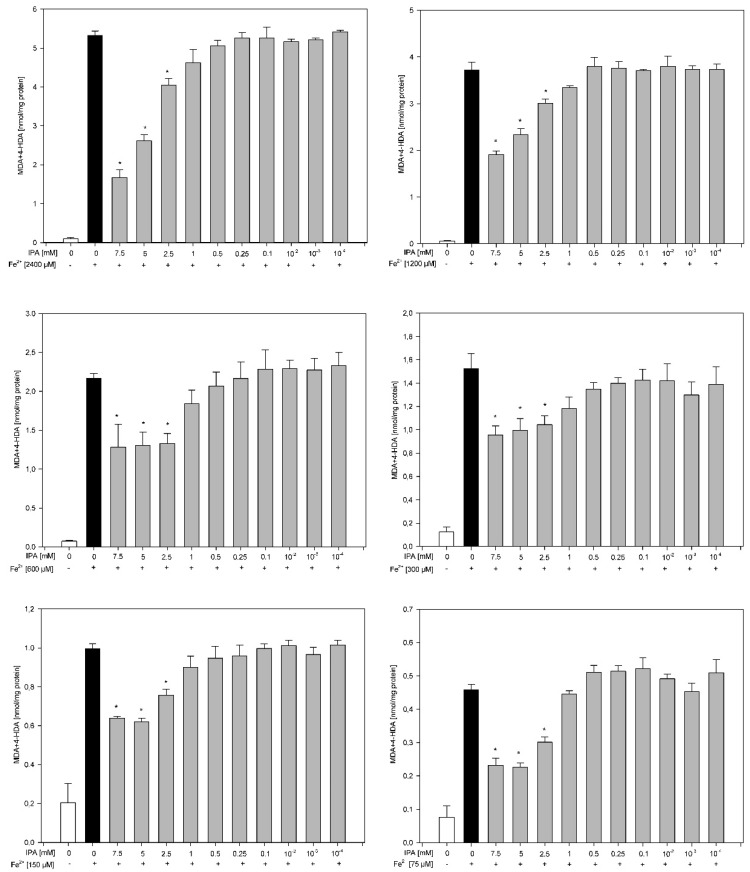
Concentration of malondialdehyde + 4-hydroxyalkenals (MDA + 4-HDA) in skin homogenates. Homogenates were incubated in the presence of FeSO_4_ used in one of six different concentrations (2400, 120, 600, 300, 150 and 75 µM) plus H_2_O_2_ (5 mM) and, additionally, in the presence of IPA (0.0, 7.0, 5.0, 2.5, 1.0, 0.5, 0.25, 0.1, 0.01, 0.001 and 0.0001 mM). Data are expressed as the amount of MDA + 4-HDA (nmol) per mg of protein. Bars represent the mean ± SE of three independent experiments run in duplicate. * *p* < 0.05 vs. the respective concentration of Fe^2+^ (black bars). All bars representing either Fe^2+^ (black bars) or Fe^2+^ plus IPA (gray bars) are significantly higher than bars representing control (white bars).

Results of the third experiment revealed that the protective effects of IPA against experimentally induced LPO did not depend on the FeSO_4_ concentration. Against all used concentrations of FeSO_4_, IPA was effective in concentrations as low as 2.5 mM, i.e., in three the highest used concentrations of 7.5 mM, 5.0 mM and 2.5 mM) (Figure 3).

## 4. Discussion

In most studies published previously, iron was used in concentrations not exceeding the value of 300 µM to induce oxidative damage in vitro [9,10,11,14,15,22]. This work is the second in which we applied ferrous in extremely high concentrations, i.e., as high as 2400 µM; the first study was performed on other tissues, namely porcine thyroid and ovary [12].

Skin iron concentrations in patients suffering from hemochromatosis have been found to be about 0.32–7.33 μmol/g dry weight tissue (0.32–7.33 mM), depending on the area of skin being examined, such as the stratum corneum or the stratum spinosum, while the physiological iron concentration in human skin is 0.06–1.77 μmol/g dry weight tissue (0.06–1.77 mM) [27]. Thus, the range of iron concentrations used in the present study (0.075–2.4 mM) corresponds to iron accumulation in the human skin under both physiological and pathological conditions, with the highest applied concentration of 2400 µM corresponding rather to concentrations found in patients suffering from hemochromatosis.

It is known that one of the characteristic symptoms of hemochromatosis is bronze skin pigmentation (hyperpigmentation) usually caused by the increased deposition of melanin in the epidermis (hypermelanosis) and of other endo- or exogenous pigments, such as iron, in the same localization. Because melanin and melatonin sound similar, the difference between these terms needs to be explained. Melatonin, as mentioned before, is a neurohormone produced mainly by the pineal gland. Melanin is a natural pigment, produced through melanogenesis in a specialized group of cells known as melanocytes. It was observed that in patients with hemochromatosis, iron can stimulate melanogenesis, but the basic mechanism of this process is unclear [28]. There is evidence that melatonin can inhibit melanogenesis and melanocyte proliferation [24]. Interestingly, melanin protects cells against the harmful effects of UV radiation and, similarly to melatonin, is responsible for the elimination of free radicals [29]. In addition, melanin content has an impact on susceptibility to oxidative stress [30]. 

It can also be mentioned that patients with vitiligo and eczema have a high serum concentration of MDA [31,32], one of the parameters of oxidative damage to membrane lipids measured in the present work.

In the current study, we observed a reduction in experimentally induced LPO due to melatonin or IPA treatment, with somewhat stronger protective effects caused by the latter. The protective effect of IPA was independent of iron concentration and was observed for IPA concentrations as low as 2.5 mM. Melatonin revealed similar preventive effects; however, it was not effective against the highest iron concentration of 2400 µM. Therefore, it should be stated that oxidative damage caused by ferrous ions in concentrations corresponding to pathological conditions was prevented by IPA but not by melatonin. Our results, namely the stronger protective effects of IPA compared to melatonin, are consistent with the results obtained by Chyan and coworkers, who demonstrated that the radical scavenging efficiency of IPA surpassed that of melatonin [33]. We also recently documented the somewhat stronger protective effects of IPA compared to melatonin against iodate-induced oxidative damage to membrane lipids in porcine thyroid [34]. However, it should be noted that results from in vitro studies may not be directly extrapolated to in vivo conditions. It should be stressed that, currently, it has not been scientifically justified to recommend IPA over melatonin in clinical situations.

The protective effects of melatonin and IPA against Fenton reaction-induced oxidative damage were confirmed in numerous previous studies [9,10,11,14,22]. In the context of the present work, it is worth mentioning our earlier results showing that oral melatonin treatment in former smokers reversed enhanced oxidative damage to membrane lipids in blood serum but not in the facial epidermis; however, it improves the biophysical characteristics of facial skin [6]. Similarly, it has been shown that melatonin reduces wrinkle formation via the inhibition of ROS [35].

The present study is the first in which skin tissue was exposed to Fenton reaction substrates under in vitro conditions. This work is also the first to document that two indole substances, i.e., melatonin and IPA, are highly protective when iron is used in extremely high concentrations corresponding to these skin iron concentrations, which are found in patients with hemochromatosis.

Taking into account the results of the present work and the above discussion, it can be suggested that two natural indole substances, i.e., melatonin and IPA, may serve as potential therapeutic agents to offset negative dermatological abnormalities associated with high iron stores in organisms or with exposure to other pro-oxidants either endogenous or exogenous. Of importance is the fact that the pro-oxidative activity of these antioxidants has never been unequivocally documented. These properties make melatonin and IPA much better antioxidants than other known free radical scavengers.

This study has certainly some limitations. The first is that our study is not an in vivo study, and we did not use whole organs; instead, we used tissue homogenates, so our results may not be directly extrapolated into in vivo conditions, especially in human populations. The other is that we used only one experimental method to measure oxidative damage to membrane lipids. The experimental method we used (a spectrophotometric assay, evaluating lipid peroxidation by measuring MDA + 4-HDA) has some disadvantages, for example the time-dependent loss of free aldehydes [36]. Although spectrophotometric methods are known to be nonspecific and can lead to an overestimation of lipid peroxidation, they are commonly used in laboratories [37].

## 5. Conclusions

Melatonin and IPA effectively protect against oxidative damage to membrane lipids induced in vitro by high concentrations of iron in porcine skin. Both antioxidants can be considered pharmacological agents in the case of disorders associated with iron overload, especially with excessive iron accumulation in the skin.

## Data Availability

Not applicable.
